# Supplementary figures and images for: Sex Identification of Feather Color in Geese and the Expression of Melanin in Embryonic Dorsal Skin Feather Follicles
Source: Animals (Basel). 2022 May 31;12(11):1427. doi: 10.3390/ani12111427 (PMC9179848; doi:10.3390/ani12111427)

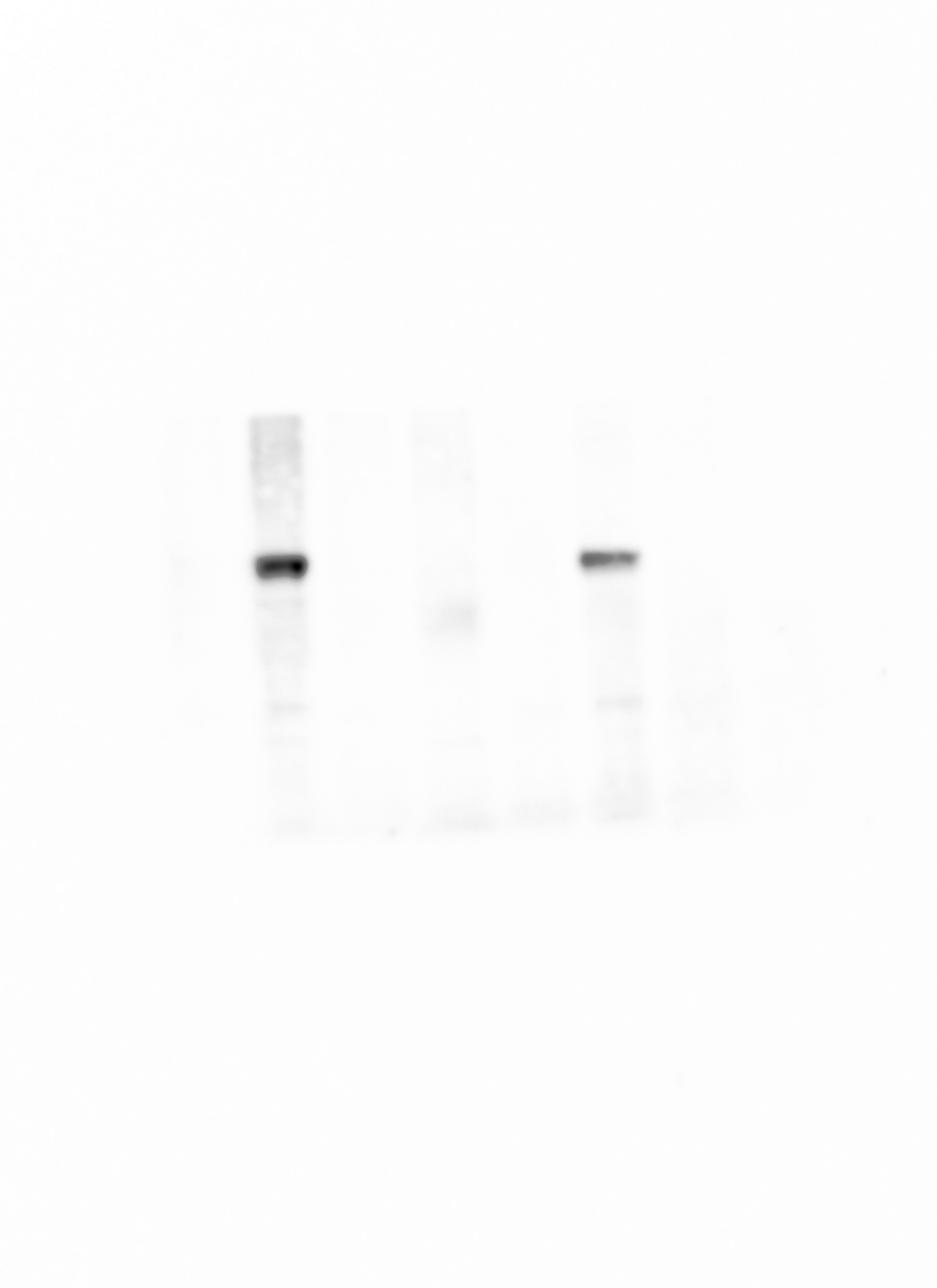

Supplement: Supplementary file 1 [file animals-12-01427-s001.zip › TYRP1 & a┬-Actin/Tyrp1 2021.11.22_00.31.25_Ch.tif]

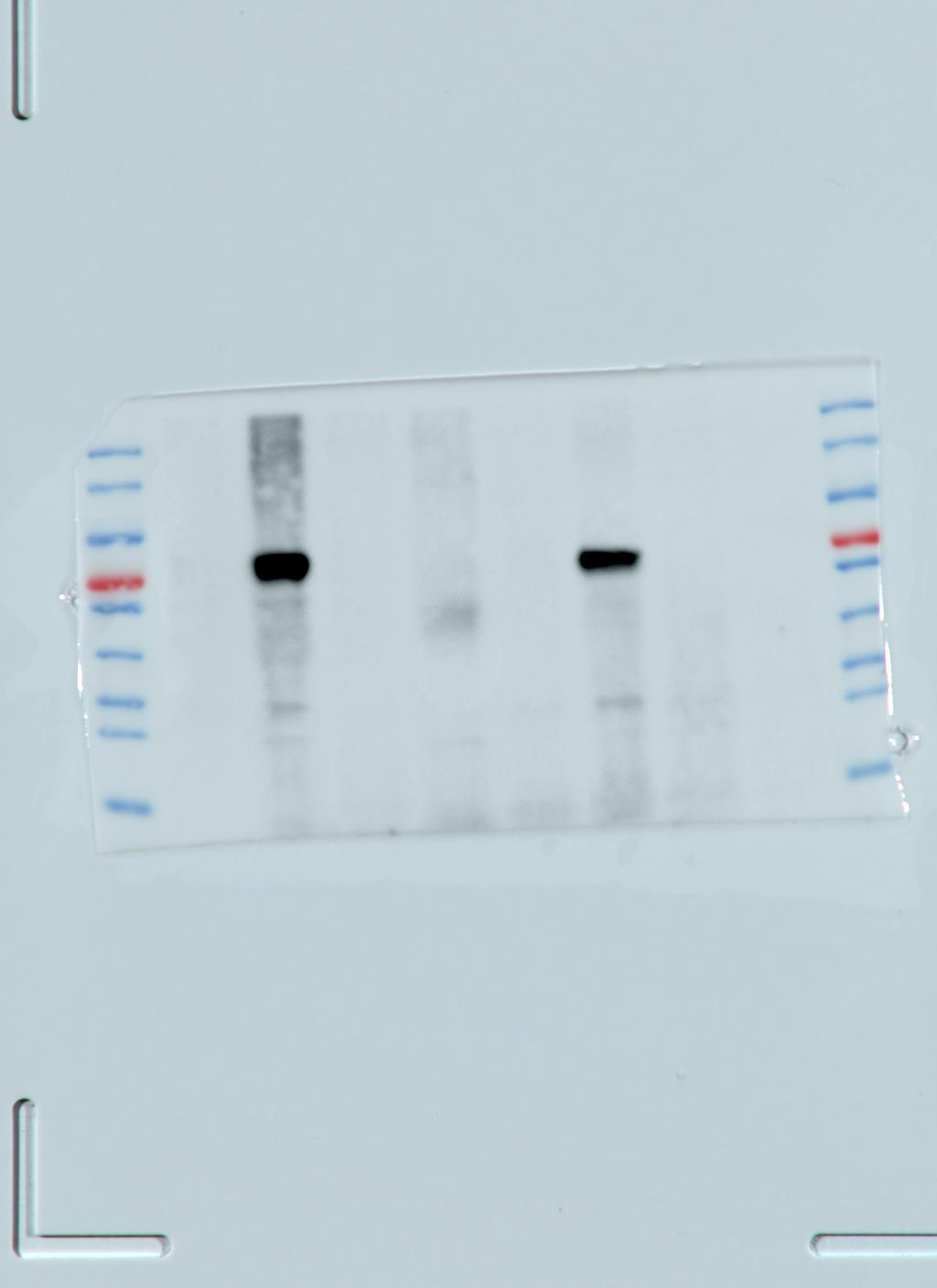

Supplement: Supplementary file 1 [file animals-12-01427-s001.zip › TYRP1 & a┬-Actin/Tyrp1 2021.11.22_00.31.25_Ch+Marker.jpg]

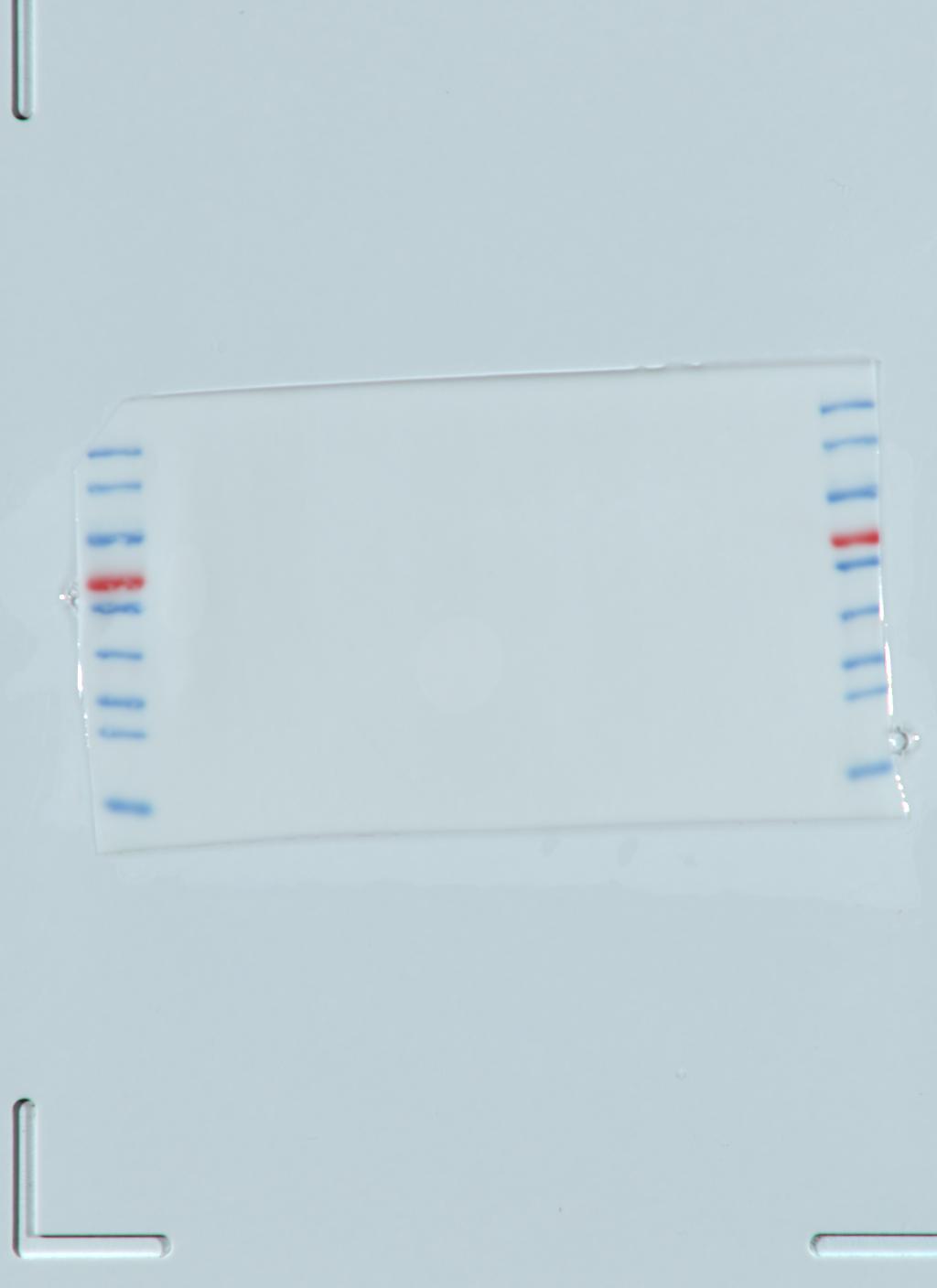

Supplement: Supplementary file 1 [file animals-12-01427-s001.zip › TYRP1 & a┬-Actin/Tyrp1 2021.11.22_00.31.25_Ch-Marker.jpg]

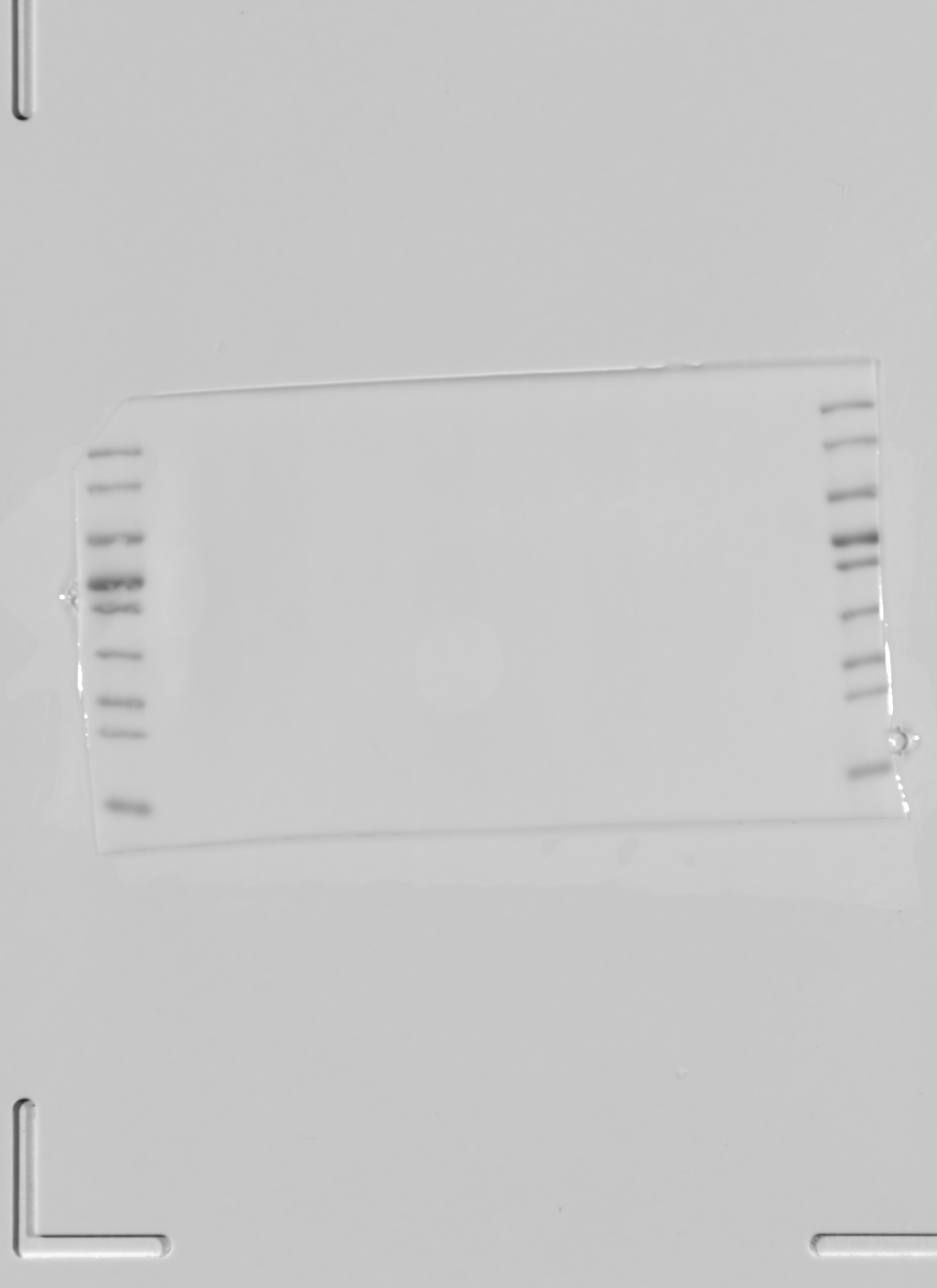

Supplement: Supplementary file 1 [file animals-12-01427-s001.zip › TYRP1 & a┬-Actin/Tyrp1 2021.11.22_00.31.25_Ch-Marker.tif]

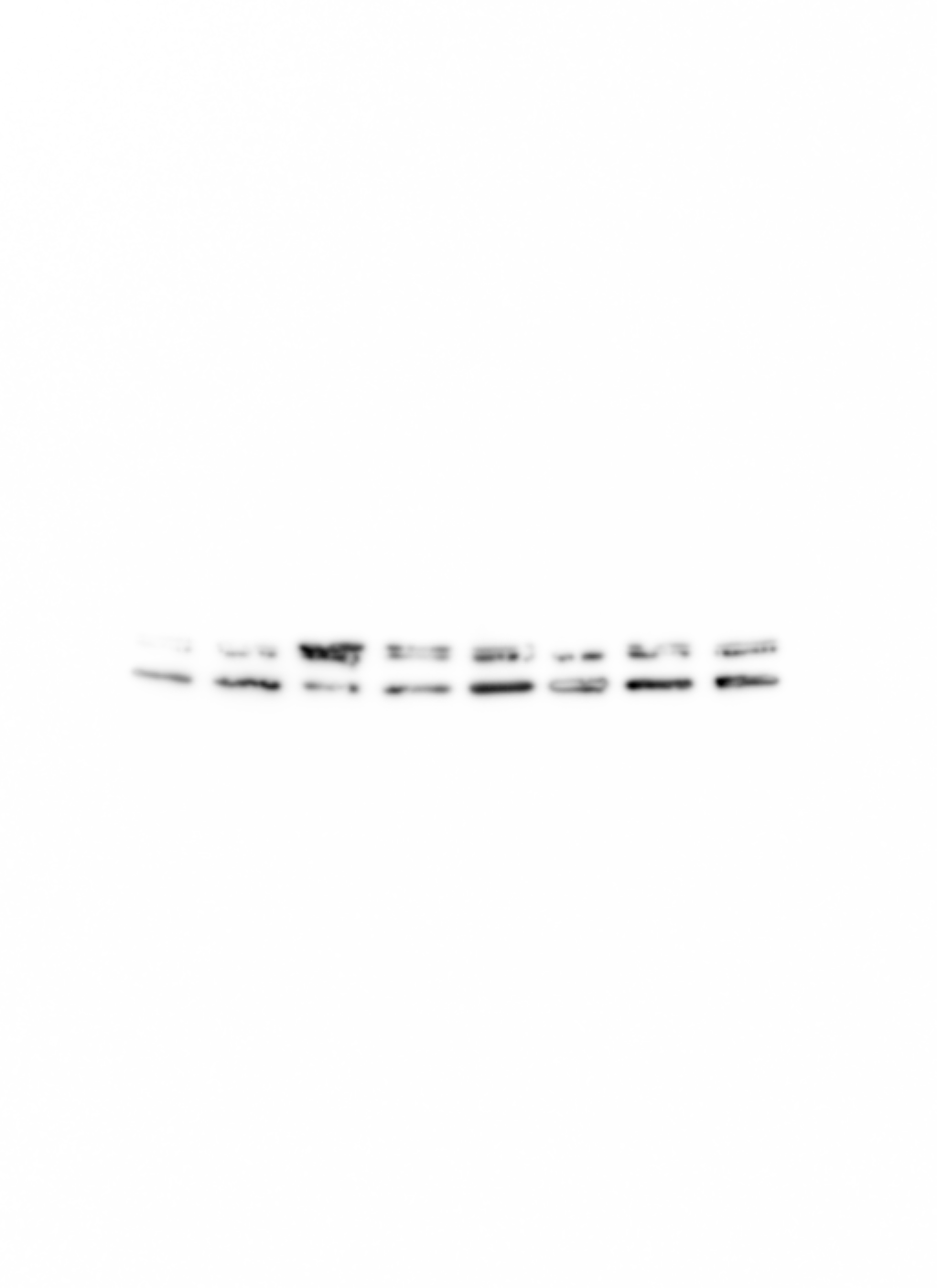

Supplement: Supplementary file 1 [file animals-12-01427-s001.zip › TYRP1 & a┬-Actin/a┬-Actin 2021.11.22_01.55.55_Ch.tif]

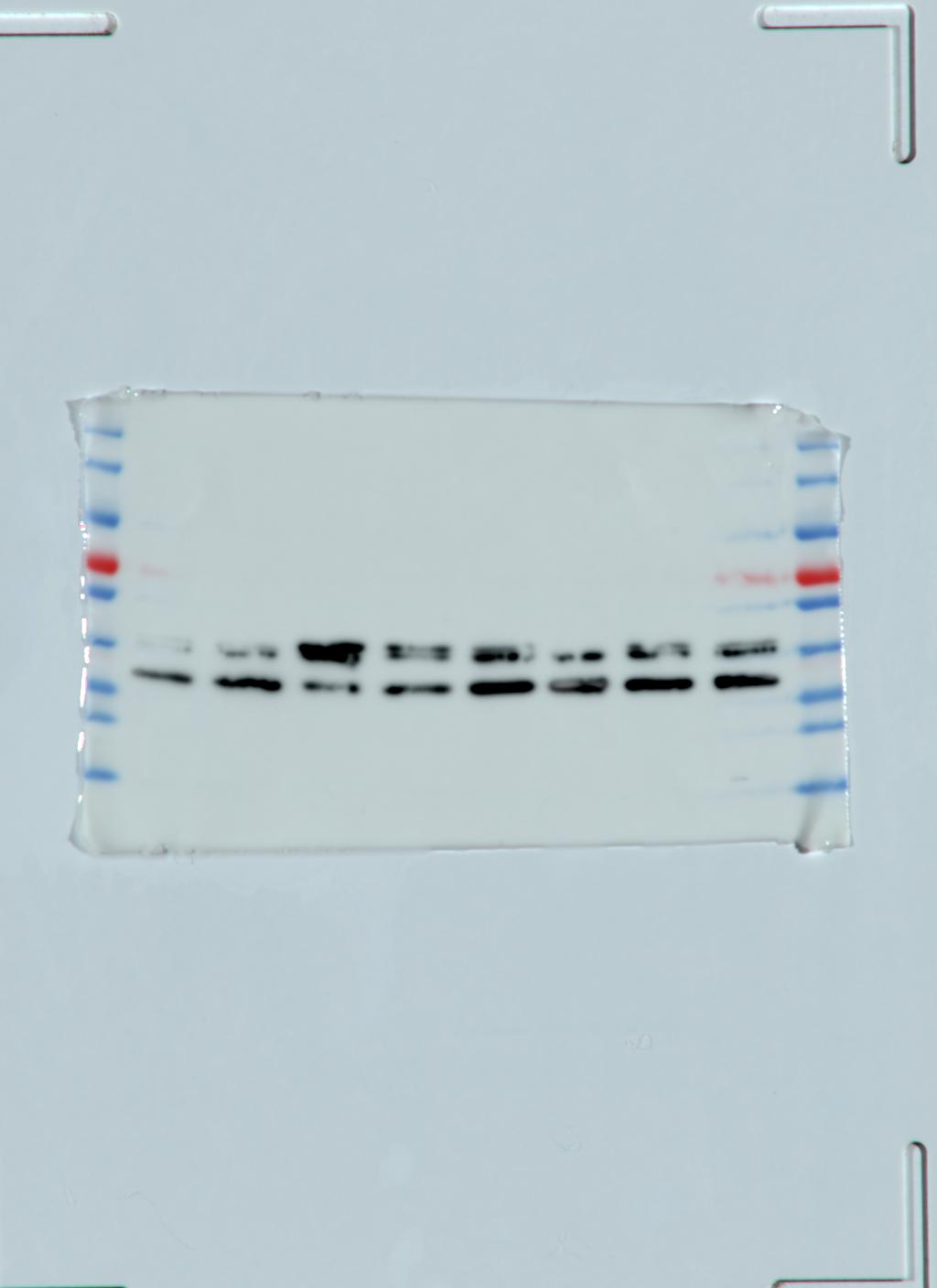

Supplement: Supplementary file 1 [file animals-12-01427-s001.zip › TYRP1 & a┬-Actin/a┬-Actin 2021.11.22_01.55.55_Ch+Marker.jpg]

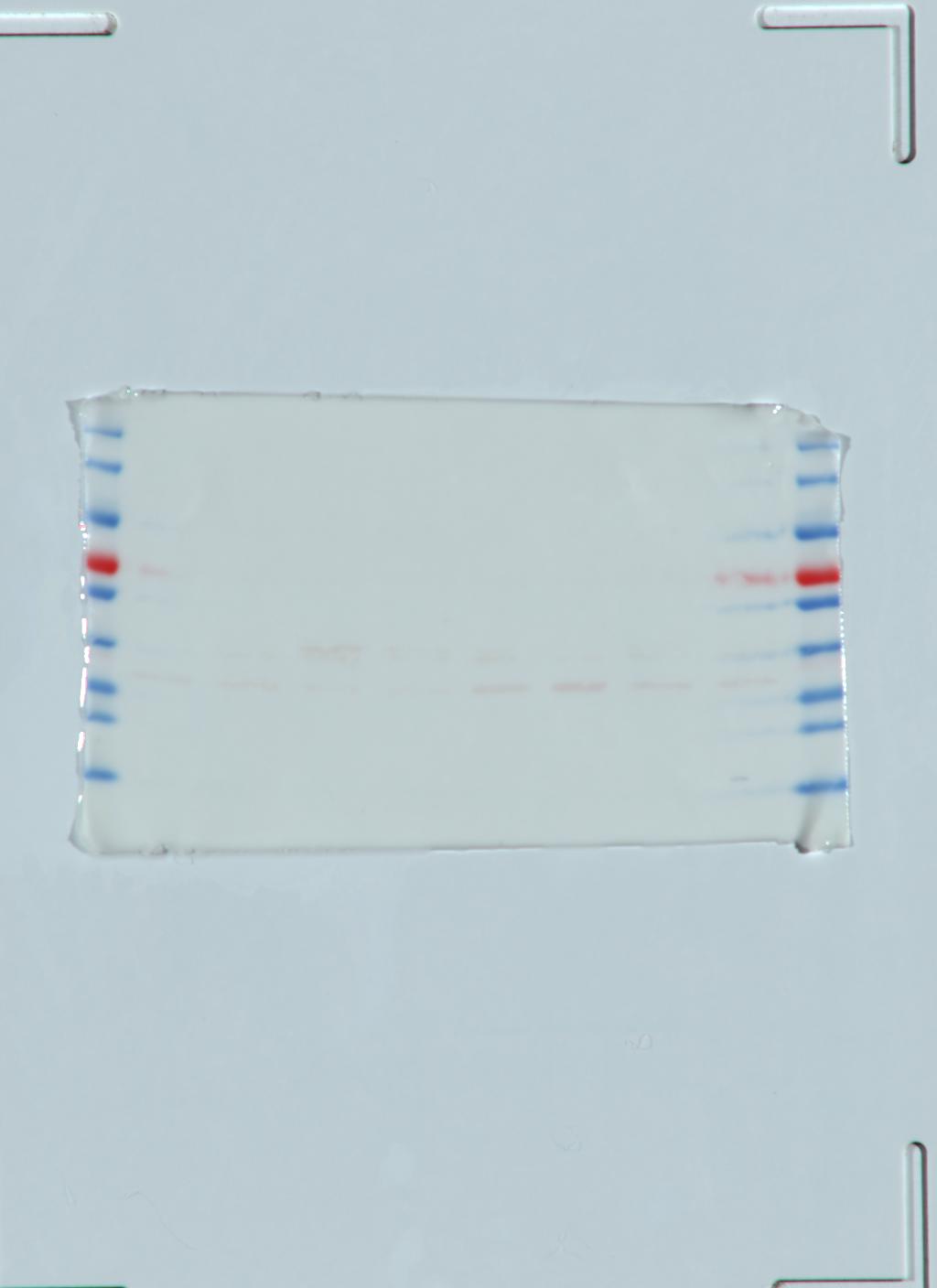

Supplement: Supplementary file 1 [file animals-12-01427-s001.zip › TYRP1 & a┬-Actin/a┬-Actin 2021.11.22_01.55.55_Ch-Marker.jpg]

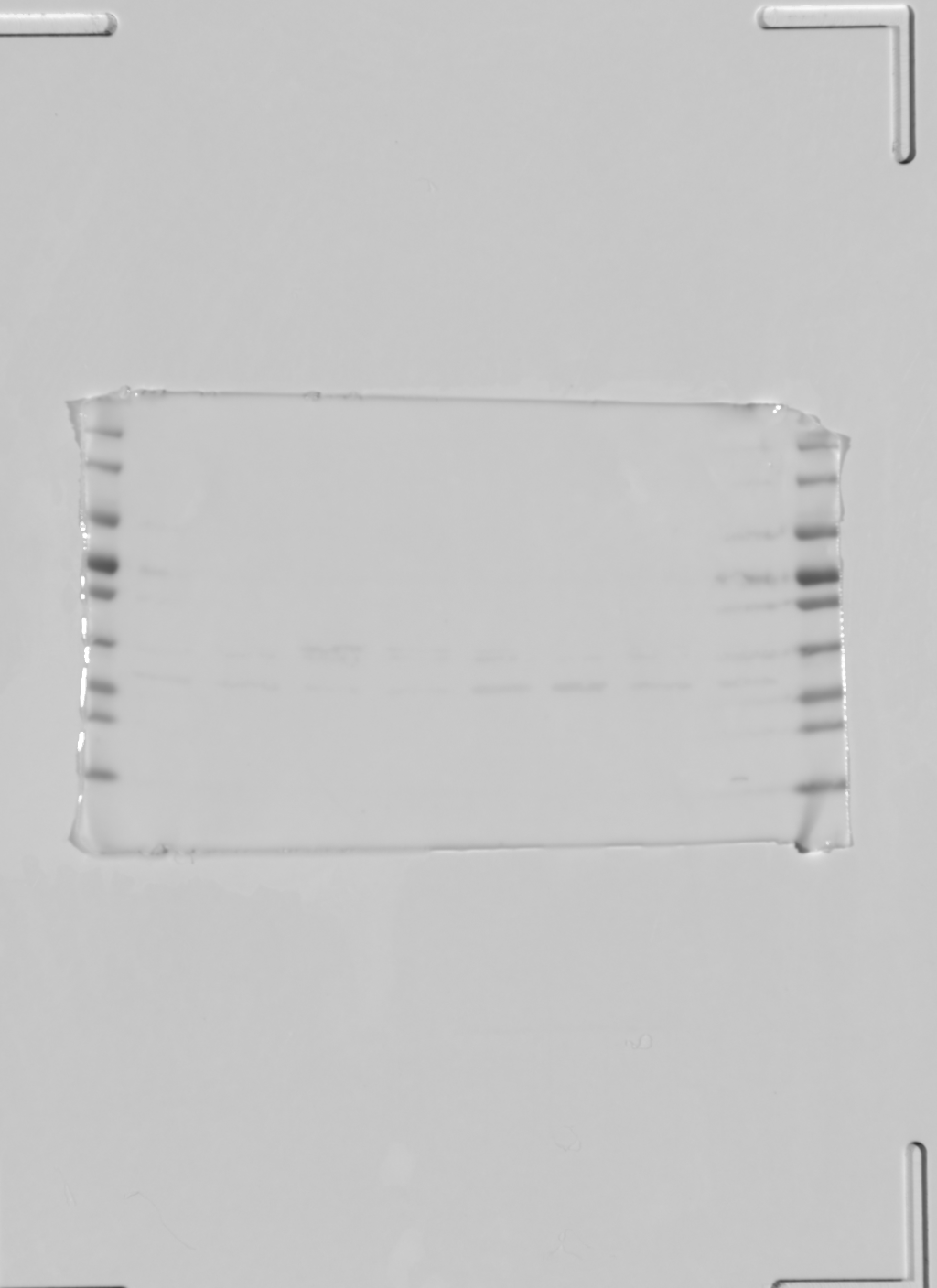

Supplement: Supplementary file 1 [file animals-12-01427-s001.zip › TYRP1 & a┬-Actin/a┬-Actin 2021.11.22_01.55.55_Ch-Marker.tif]
